# Anti-Spike Antibodies Present in the Milk of SARS-CoV-2 Vaccinated Mothers Are Complement-Activating

**DOI:** 10.3390/ijms24054395

**Published:** 2023-02-23

**Authors:** Chiara Agostinis, Miriam Toffoli, Andrea Balduit, Alessandro Mangogna, Hadida Yasmin, Chiara Ragazzon, Silvia Pegoraro, Giuseppina Campisciano, Guglielmo Stabile, Gabriella Zito, Uday Kishore, Manola Comar, Federica Scrimin, Roberta Bulla, Giuseppe Ricci

**Affiliations:** 1 Institute for Maternal and Child Health, IRCCS Burlo Garofolo, 34137 Trieste, Italy; 2 Department of Medical, Surgical and Health Science, University of Trieste, 34129 Trieste, Italy; 3 Immunology and Cell Biology Laboratory, Department of Zoology, Cooch Behar Panchanan Barma University, Cooch Behar 736101, West Bengal, India; 4 Department of Veterinary Medicine, United Arab Emirates University (UAEU), Al Ain P.O. Box 15551, United Arab Emirates; 5 Department of Life Sciences, University of Trieste, 34127 Trieste, Italy

**Keywords:** COVID-19 vaccines, antibodies, breast milk, complement system, classical pathway

## Abstract

Although only 0.8–1% of SARS-CoV-2 infections are in the 0–9 age-group, pneumonia is still the leading cause of infant mortality globally. Antibodies specifically directed against SARS-CoV-2 spike protein (S) are produced during severe COVID-19 manifestations. Following vaccination, specific antibodies are also detected in the milk of breastfeeding mothers. Since antibody binding to viral antigens can trigger activation of the complement classical - pathway, we investigated antibody-dependent complement activation by anti-S immunoglobulins (Igs) present in breast milk following SARS-CoV-2 vaccination. This was in view of the fact that complement could play a fundamentally protective role against SARS-CoV-2 infection in newborns. Thus, 22 vaccinated, lactating healthcare and school workers were enrolled, and a sample of serum and milk was collected from each woman. We first tested for the presence of anti-S IgG and IgA in serum and milk of breastfeeding women by ELISA. We then measured the concentration of the first subcomponents of the three complement pathways (i.e., C1q, MBL, and C3) and the ability of anti-S Igs detected in milk to activate the complement in vitro. The current study demonstrated that vaccinated mothers have anti-S IgG in serum as well as in breast milk, which is capable of activating complement and may confer a protective benefit to breastfed newborns.

## 1. Introduction

The complement (C) system is an innate immune surveillance system consisting of a complex network of plasma and membrane-associated proteins that are designed for the recognition and clearance of pathogens, neoplastic antigens, immune complexes, and apoptotic bodies, in order to maintain physiological homeostasis [1,2,3,4]. The C system mainly acts as an enzyme lytic cascade through three broad effector functions following the recognition of activating surfaces or ligands: opsonisation and enhancement of phagocytosis, contribution to inflammatory responses mainly via anaphylatoxins C3a and C5a, and target-cell lysis by C5b-9 membrane attack complex [5,6]. Antibody-dependent C-system activity is one of the most efficient mechanisms against infectious pathogens (e.g., bacteria, viruses, fungi, parasites). Secreted antibodies, such as IgM and/or IgG, bind to pathogens, which are recognized by the classical pathway initiation molecule C1q via the globular head (gC1q) region [7,8]. Secretory dimeric IgA (but not monomeric IgA) can also activate the C system via the lectin pathway, through the binding of mannose binding lectin (MBL), ficolins, or collectin-11 [9,10].

A number of recent studies have suggested that C-system hyperactivation, directly or indirectly by SARS-CoV-2 proteins, contributes to the pathogenesis in COVID-19 [11,12,13,14]. Specifically, reports highlighted the ability of MBL, ficolin-2, and collectin-11 to bind spike glycoprotein (S) and nucleocapsid protein (N) of SARS-CoV-2, and promote C3b and C4b deposition [15]; IgG and IgM antibodies directed against the receptor-binding domain (RBD) of S are considered as key players for the classical pathway activation [16].

An increased risk of hypertensive disorders in pregnant women who are exposed to SARS-CoV-2 in their early stage of pregnancy has been recently reported who are likely to develop pre-eclampsia [12,17]. Despite the higher risk of severe disease, the administration of COVID-19 vaccines during pregnancy was initially limited, as pregnant women were excluded from pre-authorization clinical trials due to safety concerns. Once a lack of adverse effects was demonstrated, COVID-19 vaccination was gradually extended to pregnant and breastfeeding women [18,19].

Four COVID-19 vaccines initially received authorization for emergency use by the EMA: two mRNA vaccines from Pfizer-BioNTech (BNT162b2) and Moderna (mRNA-1273), and two adenovirus-vectored vaccines from Johnson & Johnson/Janssen (Ad26.CoV2.S) and Oxford-AstraZeneca (AZD1222, Vaxzevria) [20,21]. COVID-19 vaccines incorporated S to elicit robust T-cell responses, along with high anti-viral neutralizing antibody production by B cells. Although the majority of the trials have examined the antibody responses in the blood of vaccinated and infected individuals, few studies have assessed the possible presence of anti-SARS-CoV-2 antibodies in breast milk. Notably, mother’s milk is not only the gold standard for providing nutrients (including carbohydrates, lipids, proteins, vitamins, and minerals, as well as bioactive molecules, such as cytokines, growth factors, and oligosaccharides) [22], but also the first source of antibody-mediated immune protection to the immunologically naïve and immature new-borns [7,23,24]. Human milk contains a variety of Igs, IgA being the most abundant (>90%) [25], followed by IgM and IgG [26]. Various studies evaluating S-specific Igs in the breast milk have shown high levels of IgA and IgG, and negligible IgM levels [27,28,29].

Vaccination against SARS-CoV-2 during the lactation period has been shown to significantly augment the level of antibodies in breast milk [30,31,32,33]. Interestingly, it has been reported that maternal vaccination with an mRNA-based vaccine during lactation resulted in higher SARS-CoV-2 antibody response in human milk compared to vector-based vaccines [31,34,35].

Since the possible contribution of the C system to COVID-19-related maternal immunity has not yet been examined, the present study aimed to investigate not only the presence of IgG and IgA against S in the serum and in the milk of SARS-CoV-2-vaccinated breastfeeding healthcare and educational workers, but also their capability to activate the C system.

## 2. Results

### 2.1. Determination of the Anti-Spike-Specific Antibody Titre in Serum and Milk of Vaccinated Mothers

First, we tested for the presence of anti-S IgG and IgA in serum and milk using a cohort of 22 SARS-CoV-2-vaccinated women (*n* = 17 with Pfizer–BioNtech, *n* = 4 with Oxford–AstraZeneca, *n* = 1 with both vaccines; Table S1). In order to consistently compare IgG with IgA levels, as well as the values obtained for serum and milk samples, we used an immune serum pool (COVID-19-recovered and -vaccinated patients) as a standard calibrator. The calibrator was previously titred, obtaining a 1:75,000 titre for both IgG and IgA (Table S2). To determine this titre, plates were coated with S, then serum (1:50–1:200 dilution) and milk samples (1:2–1:4 dilution) were incubated and the binding of Igs was detected using specific anti-human IgG, anti-human serum IgA, or anti-human secretory IgA. A milk pool showed high positivity for IgA antibodies, thus it was used as a calibrator for secretory IgA.

**Figure 1:**
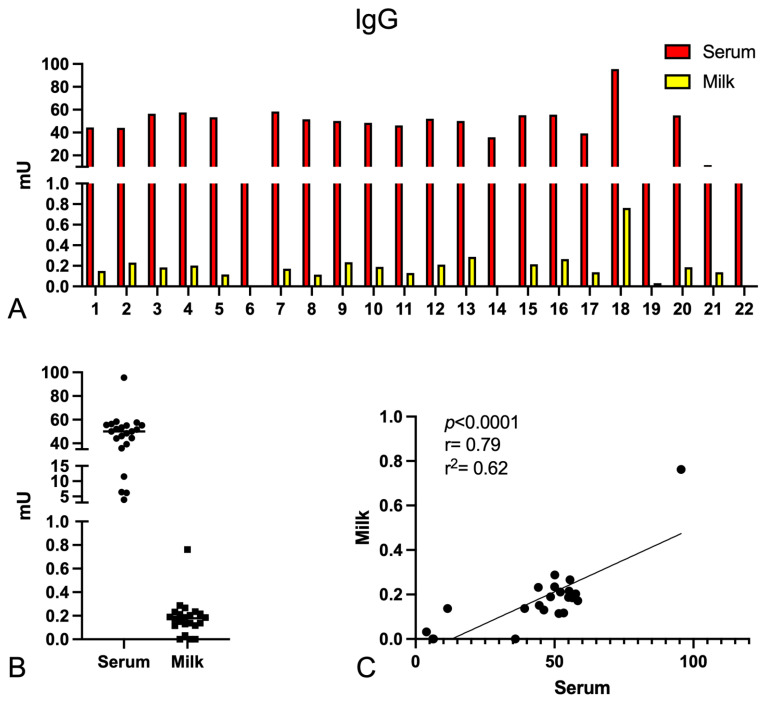

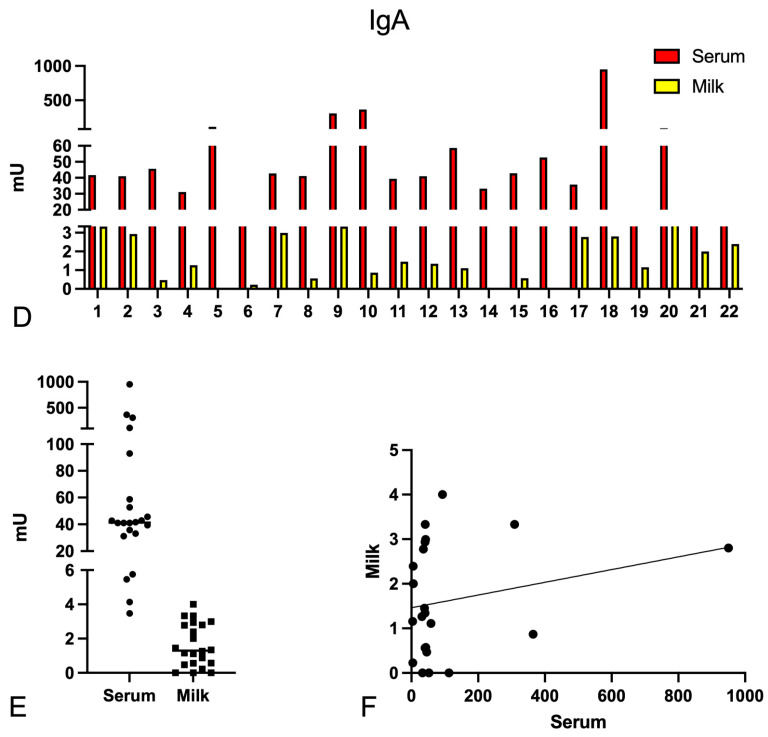
Measurement of anti-Spike IgG and IgA levels in serum and milk of vaccinated and lactating women. Serum and milk samples of the enrolled women (*n* = 22) were quantified for the presence of anti-S IgG (**A**–**C**) and IgA (**D**–**F**). Despite a wide variability, Ig levels appeared to be greater in the serum as compared to the milk (**B**,**E**). A statistically significant correlation was found between serum and milk IgG levels (Pearson test, *p* < 0.0001, r^2^ = 0.62) (**C**), whilst no correlation was found for IgA (**F**).

As shown in Figure 1, patients presented a wide range of IgG and IgA levels in serum as well as milk, although the Ig levels were 200–300 times higher in serum than in breast milk. The correlation (Pearson test) between serum and milk Ig levels revealed a statistically significant value (*p* < 0.0001, r^2^ = 0.62) for IgG, whereas no statistical significance was obtained for IgA levels. Surprisingly, no correlation was observed between Ig levels and days elapsed between vaccination and sample collection (Figure S1). By identifying patients with low IgA levels, we noticed that almost all were vaccinated with Oxford–AstraZeneca (patients #2, #4, #6, #21, and #22), while patients displaying very high levels of IgA had previously contracted SARS-CoV-2 (patients #18, #19, and #20).

### 2.2. Evaluation of C1q, MBL, and C3 Levels in the Serum and Milk of Vaccinated Mothers

**Figure 2:**
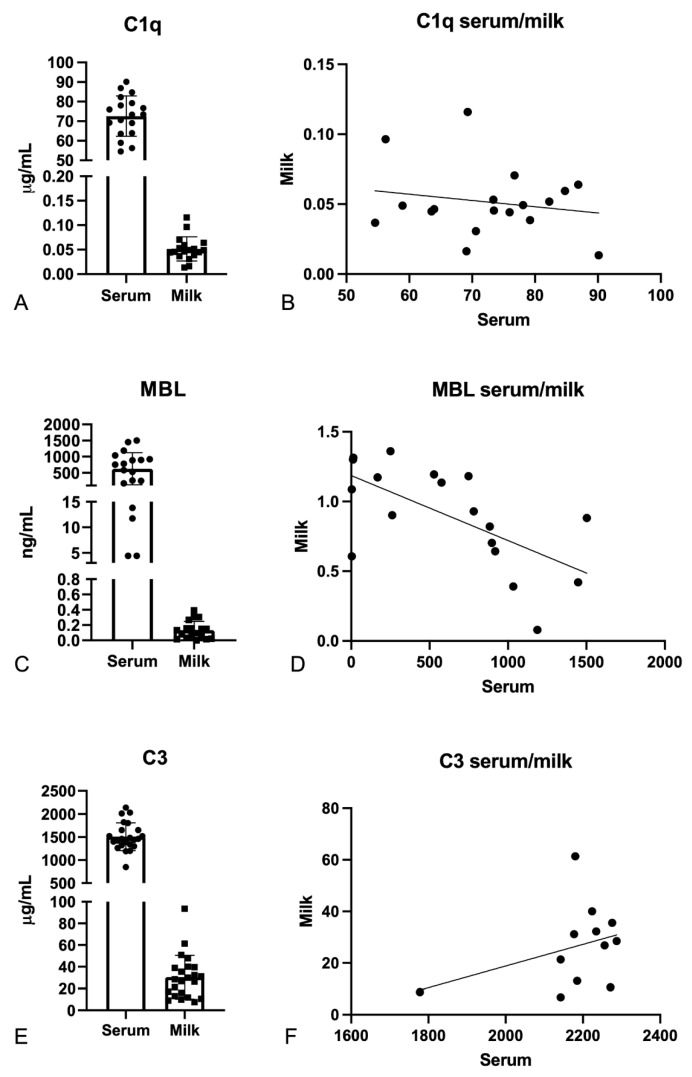
C1q, MBL, and C3 levels in serum and milk of vaccinated women. The levels of the three complement pathway activators (C1q, MBL, and C3) were evaluated using commercial ELISA kits. C1q and MBL levels appeared to be higher in serum than in milk samples (**A**,**C**); however, no correlation was found between the two biological fluids (**B**,**D**). C3 measurement revealed an abundance of this complement activator in milk, although the level remained lower than in serum (**E**). No correlation was found between C3 serum and milk concentrations (**F**).

Since the main aim of this study was to understand whether the presence of anti-S antibodies in breast milk after anti-SARS-CoV-2 vaccination may be responsible for C-system activation, we first analyzed the levels of C1q, MBL, and C3, the first recognition subcomponents of the classical, lectin, and alternative pathways, respectively (Figure 2). Thus, we measured their levels in milk and sera of our cohort of patients using commercial ELISA kits. Our results indicated that the C1q level in milk was about 500–1000 times lower than that in serum; MBL, when physiologically present, was about 3000–5000 times lower in milk than in serum; and C3 appeared to be only 20–70 times lower in the milk than in serum. For all three subcomponents, we could not establish a statistically significant correlation between their serum and milk levels (Figure 2B,D,F). Then, we evaluated complement C1q, MBL, and C3 levels in the milk of our cohort of patients via Wieslab ELISA assay, a test designed for analyzing C-system functionality in the serum. Milk samples failed to validate activation of all the three C-system cascades, whilst all the sera were found to be C-sufficient (Figure S2).

### 2.3. Complement Activation by Igs Anti-Spike Antibodies Present in Serum and Milk

**Figure 3:**
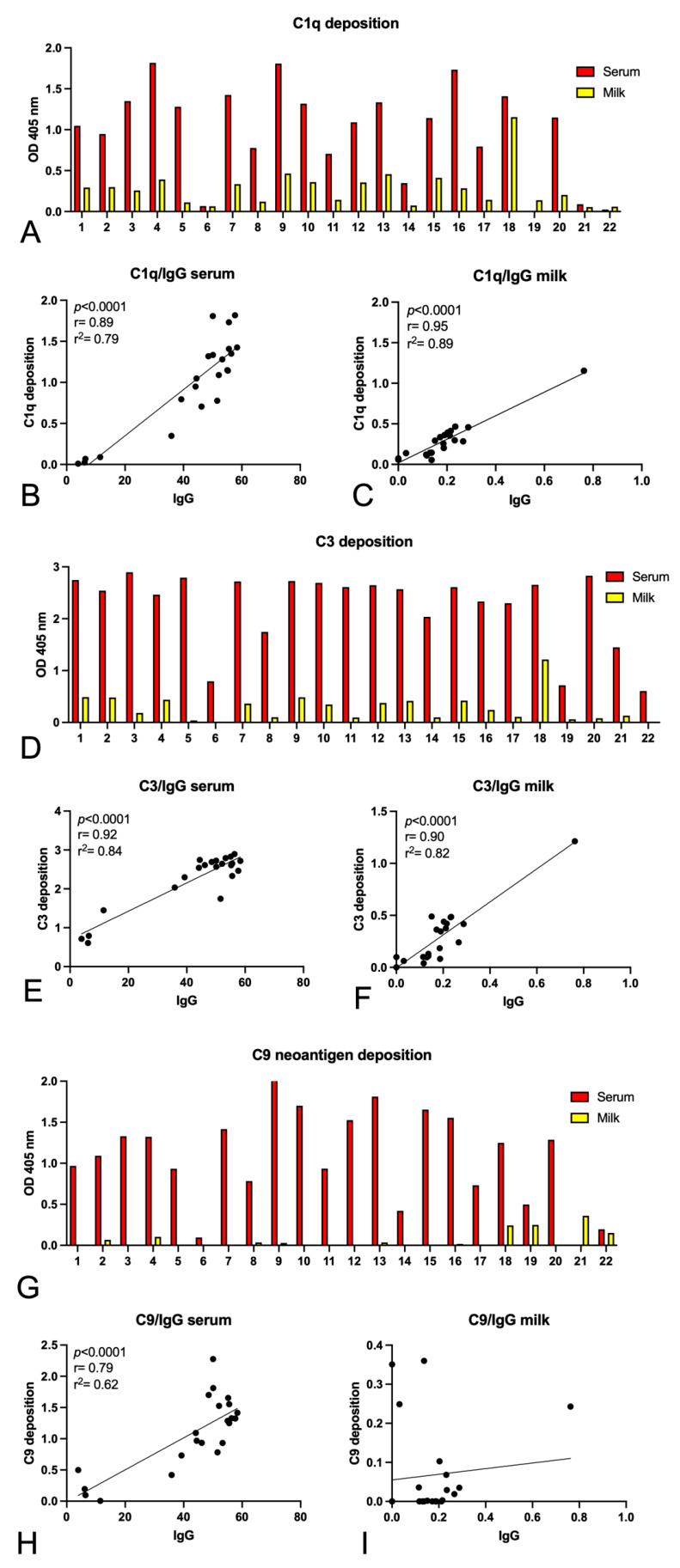
Complement activation by anti-S Igs present in the serum and the milk of vaccinated women. C-system activation by anti-S Igs of serum and milk was evaluated by ELISA for the detection of C1q (**A**–**C**), C3 (**D**–**F**), and C9 neoantigen deposition (**G**–**I**).

Finally, we investigated the ability of anti-S Igs present in the milk of vaccinated women to activate the C system in vitro. Following the binding of serum or milk IgG to S, a pool of AB Rh+ sera was added to the wells as a source of C-system components, and subsequently, the deposition of C1q, C3, and C9 neo-antigens was detected by adding specific antibodies (Figure 3). In addition, a wide variability of C-system activation components was also observed; the milk samples identified for their ability to strongly activate the C system were obtained independently from patients having received Pfizer–BioNTech or Oxford–AstraZeneca vaccine or being COVID-recovered. Almost all the milk samples were able, even though at low levels, to induce C1q (Figure 3A) and C3 (Figure 3D) deposition, but only few samples led to the activation of the whole cascade until the formation of the C9 polymer (Figure 3G).

We observed a significant correlation between C1q or C3 deposition and IgG presence, both in serum and in milk (Figure 3B,C,E,F), whereas no correlation for C9 neoantigen deposition was found in milk (Figure 3H,I).

**Figure 4:**
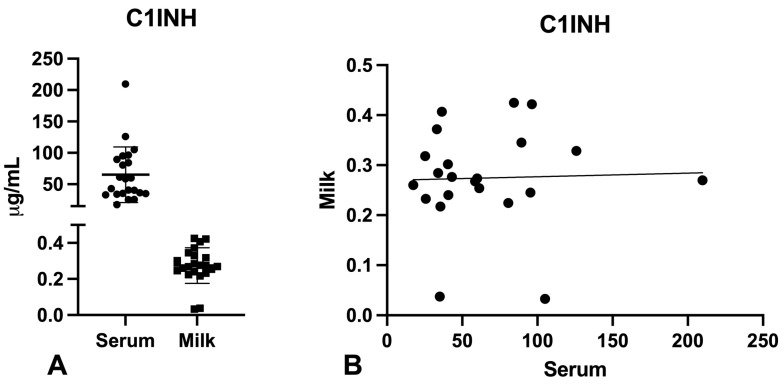
Evaluation of C1 inhibitor (C1INH) levels in serum and milk samples. C1INH levels were quantified by using a commercial ELISA kit (**A**). C1INH levels in milk and serum were correlated by Pearson test. No correlation was found between serum and milk concentrations (**B**).

Since the activation of the classical pathway, induced by the binding of specific anti-S antibodies to their target, could be potentially blocked by the activity of C1-inhibitor, we also evaluated the presence of this protein in the serum and milk of the enrolled women. We found high levels of this C-system inhibitor in breast milk compared to the other C-system components previously measured, although levels were around 100–200 times lower than in the serum (Figure 4).

## 3. Discussion

During the COVID-19 pandemic, a number of studies aimed to characterize the protective antibody-mediated viral neutralization in response to SARS-CoV-2 infection in order to unveil the mechanisms of SARS-CoV-2–host interaction, mainly focusing on host immune response and C-system activation. Garred and co-workers reported that the anti-SARS-CoV-2 (RBD) IgG response in convalescent plasma was mainly driven by IgG1 and IgG3 subclasses, the main ligands for C1q-mediated activation of the classical pathway [36]. Furthermore, they observed that C4b, C3bc, and C9 polymer deposition due to antibodies specifically directed against SARS-CoV-2 could be significantly correlated with both IgG levels and disease severity, suggesting that patients with high IgG levels or severe symptoms may exhibit a more powerful C-system activation during viral infection [36]. Moreover, Cunningham and colleagues demonstrated that C1q binding to SARS-CoV-2 Igs in vitro strongly correlated with antibody responses, whereas the detection of downstream C-system components (C4b, C3b, and C5b) showed some variability depending on the group analyzed. In particular, the deposition of C3b–C5b on S was consistently observed in convalescent hospitalized patients, but not in the non-hospitalized group [37].

Interestingly, a few studies also investigated the presence of anti-S specific Igs in the milk of vaccinated mothers [27,30,31,32,33,34,35,38,39,40,41], but they did not assess if these antibodies were complement-activating. A recent study demonstrated a robust secretion of SARS-CoV-2-specific IgA and IgG in the breast milk for 6 weeks following vaccination [42]; similar findings were reported in women who had recovered from COVID-19 [33,43].

**Figure 5:**
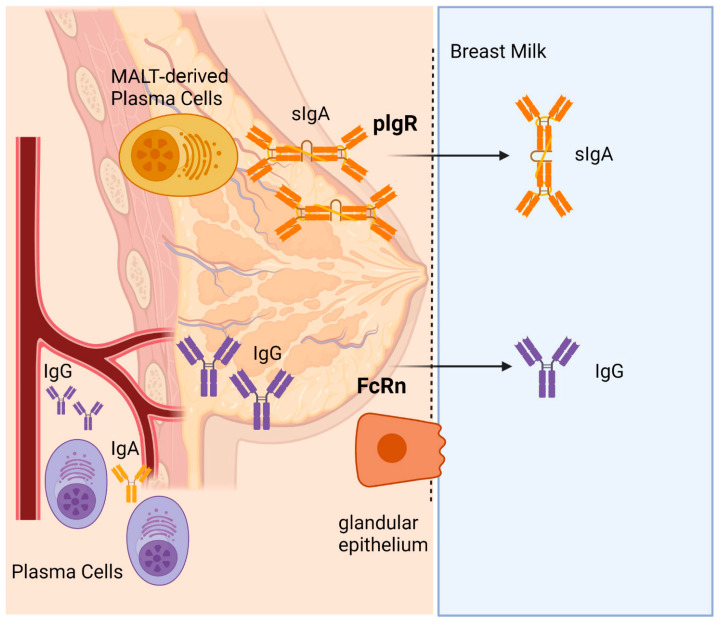
Transfer of Immunoglobulins (Igs) to the breast milk. Mucosa-associated lymphoid tissue (MALT)-derived plasma cells arise from mucosal tissues of both gut and airways and home to the mammary gland. These plasma cells produce secretory IgA (sIgA), which are passed into breast milk via the polymeric Ig receptor (pIgR) present on the mammary epithelium. IgG originating from secondary lymphoid organs and bone-marrow-derived plasma cells are transferred from the blood to the breast milk via neonatal Fc receptor (FcRn) expressed on the mammary epithelium cells.

In the current study, we initially confirmed the presence of anti-S specific IgG and IgA in serum as well as in milk samples and observed a range of antibody titres among vaccinated women. Consistent with an earlier study [44], we found that almost all women/participants with low IgA levels had received the Oxford–AstraZeneca vaccine, whereas individuals with very high levels had previously contracted COVID-19. As expected, only serum and milk IgG levels showed a statistically significant correlation, since they are both produced by the same plasma cells in secondary lymphoid organs and bone marrow whilst serum and secretory IgA are characterized by different plasma-cell origins (i.e., bone marrow for serum IgA and mucosa-associated lymphoid tissue for secretory IgA) [45,46,47,48], as summarized in Figure 5.

Surprisingly, no correlation was observed between Ig levels and number of days post-vaccination, a result assuring us that samples collected at different timepoints could be comparable. In fact, the persistence of neutralizing antibodies following COVID-19 vaccination is currently under investigation. With respect to IgA, this may be explained by studies showing that IgA levels in milk did not rise further when measured after 18 days following the second dose of vaccine, in contrast to the significant increase in IgG levels after the second immunization [49].

Antibodies elicited by mRNA-1273 vaccine were detectable in the serum until six months [50] and by AZD1222 until three months [51], whilst Ad26.COV2.S and BNT162b2 vaccines have been shown to give shorter duration protection [52]. Despite also providing consistent T-cell mediated immune responses, BNT162b2 vaccine induced anti-S IgG production 11 days after the first dose administration, showing the peak at day 21, whereas the AZD1222 elicited a neutralizing effect 22 days after the first dose [53].

Historically, very little information is available about the importance of the C system and its contribution to mucosal immunity in human breast milk, mainly due to the low levels of C-system components detected in mature milk [26]. Moreover, the relative contribution of C-system components transported from the serum and those that are locally produced is still poorly understood [54].

In order to explore potential activation of the C system in breast milk due to immunization against COVID-19, we subsequently examined the amounts of C1q, MBL, and C3, the first recognition subcomponents of the classical, lectin, and alternative pathways, respectively. Our findings showed that C1q levels in the milk were 500–1000 times lower than those in serum, and MBL levels, when physiologically present, were 300–5000 times lower. C3 was the most abundantly detected among C-system activators, being only 20–70 times less abundant in the milk than in serum samples. Despite the detection of C-system activators, even though at very low levels, we failed to demonstrate the capability of C-system components present in the milk samples to activate C cascades using Wieslab assay. This may have been due to the relatively small amount of C components present in breast milk compared to serum, highlighting the need for a more sensitive assay. Moreover, some C-component concentrations in human milk seemed to be quite similar to those detectable in the serum when considering colostrum and early lactational milk, but they significantly fell during the first few months of breastfeeding [55].

To overcome these technical limitations, we investigated the ability of anti-S Igs, previously detected in the milk of vaccinated women, to activate the C system in vitro. Even though at low levels, nearly all milk samples caused C1q and C3 deposition, but only a limited number of samples induced the formation of C9 polymer. Since in vitro activation of the C system leading to C3b deposition on killed bacteria has been already documented in human milk [56], it is tempting to hypothesize that C-system activation may mainly exert effector functions of opsonization.

Interestingly, we observed a significant correlation between C1q or C3 deposition and IgG presence, both in serum as well as milk, whereas no correlation was noticed for C9 deposition in milk. In accordance with Lamerton et al. [37], we observed that patients presenting antibodies able to induce C9-deposition in the milk belonged to the COVID-19 recovered group (#18 and #19) and, surprisingly, to the AstraZeneca-vaccinated group (#4, #21, and #22).

Even though an excessive or uncontrolled C-system activation has already been established as a well-known pathogenic player in severe COVID-19, the potential involvement of C-system factors in protective immunity against SARS-CoV-2 has been largely neglected. Since human milk represents a vehicle to transfer maternal immunity against infections to infants via bioactive factors, it is likely that Igs contained in the milk of vaccinated women maintain their ability to activate the C system along respiratory mucosa and the gastrointestinal tract of the breast-fed newborns, ensuring protection against COVID-19. This study sheds light on the possible physiological and protective significance of the C system in vaccination-driven maternal immunity.

## 4. Materials and Methods

### 4.1. Patient Enrolment and Sample Collection

The study cohort comprised twenty-two breastfeeding healthcare professionals and school workers who received the COVID-19 vaccine and were randomly selected from the cohort previously described by Scrimin et al. [41] (Table 1). Between 1 February and 30 July 2021, women experiencing physiological pregnancies and normal early postpartum in and around Trieste (Italy) were recruited by a perinatal study group at the Institute for Maternal and Child Health IRCCS “Burlo Garofolo” (Trieste, Italy). Nasopharyngeal swabs for SARS-CoV-2 testing were performed on all the enrolled mothers and newborns at the time of enrolment, one week prior to enrolment, and one week following enrolment. At the time of sample collection, the participants had no symptoms, and all the tests were found to be negative. Serum and milk samples were collected from each woman (maximum 75 days after vaccination). After nearly complete breast expression, milk samples were obtained, including the foremilk and the hindmilk. Serum and milk samples were both immediately delivered to the laboratory for storage and analysis. To separate the cells from the fat content of the milk, samples of breast milk were centrifuged at 800× *g* for 10 min at 4 °C.

The study was approved by the Internal Review Board of the Institute for Women and Child Health IRCCS “Burlo Garofolo” (IRB 06/2021). All participants signed detailed, informed consents and were over the age of 18.

### 4.2. Quantification of Anti-Spike IgG and IgA by Enzyme-Linked Immunosorbent Assay (ELISA)

For the Elisa assay, 96-well plates were first coated with 2 µg/mL SARS-CoV-2 S recombinant protein (S1/S2) (RP-87671, Invitrogen, Waltham, MA, USA) diluted in phosphate-buffered saline (PBS) and incubated overnight at 4 °C. Plates were then washed three times with PBS + 0.1% Tween 20 (PBS-T) and blocked with 3% *w/v* skimmed milk in PBS-T for 1 h at RT. Patient sera (1:200) and milk (1:2) diluted in PBS-T + 1% *w/v* skimmed milk were added and incubated 2h at RT. After washing three times, antibodies for Ig detection were added for 1 h at RT: anti-human IgG-alkaline phosphatase (AP)-conjugate (1:50,000, Sigma Merck, St. Louis, MO, USA), anti-IgA antibody (1:700, Sigma Merck) for serum samples and anti-IgA secretory component antibody (1:250, Sigma Merck) for milk samples. Anti-rabbit IgG AP-conjugate (1:10,000, Sigma Merck) and anti-mouse polyvalent Igs (G,A,M) AP-conjugated (1:30,000, Sigma Merck) were incubated 1 h at RT for the detection of anti-IgA and anti-secretory IgA, respectively. The binding of secondary antibodies was revealed using p-nitro phenyl phosphate (pNPP) as a substrate. The absorbance was read at 405 nm by PowerWave X Microplate Reader (Bio-Tek Instruments, Winooski, VT, USA). A standard curve was also included. 

### 4.3. Measurement of C1q, C3, and MBL Levels by Enzyme-Linked Immunosorbent Assay (ELISA)

Commercial ELISA kits were used to quantify C1q (Hycult Biotech, Uden, The Netherlands, #HK356-02), MBL (Hycult Biotech, #HK323), C3 (Abcam, Cambridge, UK, #ab108823), and C1 inhibitor (R&D Systems, Minneapolis, MN, USA, #DY2488-05) in serum and milk samples, following the instructions provided by the manufacturer. The absorbance was read by a PowerWave X Microplate Reader (Bio-Tek Instruments) spectrophotometer.

### 4.4. Evaluation of Complement Pathway Functionality in Serum and Milk

The functionality of classical, alternative, and lectin pathways was determined using the commercial kit Wieslab^®^ (Technogenetics, Milan, Italy). The assay was performed following the manufacturer’s instructions, with the exception of milk dilution (1:2).

### 4.5. Evaluation of Complement Activation

Patient sera (1:50) and milk (1:2) were incubated in microplate wells coated with SARS-CoV-2 S recombinant protein (S1/S2), as described above. In order to assess the capability of anti-SARS-CoV-2 antibodies to activate the C system, wells were then incubated with AB Rh+ pooled sera (1:100 in PBS + 2% *w/v* BSA + 0.7mM Ca^++^Mg^++^) for 30 min at 37 °C. After washing with PBS-T, the binding of C1q was evaluated using rabbit anti-human C1q polyclonal antibody (1:2000, Dako, Santa Clara, CA, USA) for 1 h at 37 °C and anti-rabbit IgG AP-conjugated (1:10,000, Sigma-Aldrich, St. Louis, MO, USA) as a secondary antibody. Simultaneously, the deposition of C3 was detected using goat anti-human polyclonal antibodies (1:5000, Quidel, San Diego, CA, USA) for 1 h at 37 °C and anti-goat IgG AP-conjugated (1:30,000, Sigma-Aldrich). The formation of the terminal C complex C5b-9 was assessed using anti-human C5b-9 (1:50, clone: aE11, Dako) for 1 h at 37 °C and anti-mouse polyvalent Igs (G,A,M)-AP (1:30,000, Sigma Merck). The binding was revealed with pNPP and the absorbance was read at 405 nm using a PowerWave X Microplate Reader (Bio-Tek Instruments).

### 4.6. Statistical Analysis

The experiments were run in duplicate and data are expressed as mean of values. The correlation was analyzed using the Pearson test and *p*-values < 0.05 were considered statistically significant. All statistical analyses were performed using GraphPad Prism software 9.0 (GraphPad Software Inc., La Jolla, CA, USA).

## 5. Conclusions

After infection and vaccination, the presence of anti-SARS-CoV-2 antibodies in breast milk may offer a potentially protective benefit for the nursing infant, not only for the direct presence of anti-S antibodies but also for their C-system activation capability. In general, the level of IgG antibodies in serum was higher than that in breast milk but the C-system activation potential was retained in the case of milk antibodies. Furthermore, our study raises a cardinal point that the remit of vaccine efficacy should not exclusively rely on the antibody titers and highlights the importance of describing the complex immune response in its entirety.

## Figures and Tables

**Table 1:** Characteristics of the study cohort.

Variables	*n* = 22
Age, mean (±SD)	36 (±3)
Previous COVID-19 infection, *n*	3
Vaccine type Pfizer–BioNtech, *n*	17
Vaccine type Oxford–AstraZeneca, *n*	4
Vaccine type Oxford–AstraZeneca + Pfizer–BioNtech, *n*	1
Days between collection and the 2nd dose of vaccine, mean (±SD)	27 (±13)

Abbreviations: SD, standard deviation.

## Data Availability

The original contributions presented in the study are included in the article, further inquiries can be directed to the corresponding author.

## Supplementary Materials

The following supporting information can be downloaded at: https://www.mdpi.com/article/10.3390/ijms24054395/s1.
