# Supplementary material for: Anti-Spike Antibodies Present in the Milk of SARS-CoV-2 Vaccinated Mothers Are Complement-Activating
Source: Int J Mol Sci. 2023 Feb 23;24(5):4395. doi: 10.3390/ijms24054395 (PMC10002545; doi:10.3390/ijms24054395)
Supplement: Supplementary file 1 [file ijms-24-04395-s001.zip › ijms-2161109-supplementary.pdf]

International Journal of *Molecular Sciences*

MDPI

Supplementary Material to

Anti-Spike Antibodies Present in the Milk of SARS-CoV-2 Vaccinated Mothers Are Complement-Activating

Chiara Agostinis [1,*,†], Miriam Toffoli [2,†], Andrea Balduit [1], Alessandro Mangogna [1], Hadida Yasmin [3], Chiara Ragazzon [2], Silvia Pegoraro [1], Giuseppina Campisciano [1], Guglielmo Stabile [1], Gabriella Zito [1], Uday Kishore [4], Manola Comar [1,2], Federica Scrimin [1], Roberta Bulla [5,‡,*] and Giuseppe Ricci [1,2,‡]

1 Institute for Maternal and Child Health, IRCCS Burlo Garofolo, 34137 Trieste, Italy
2 Department of Medical, Surgical and Health Science, University of Trieste, 34129 Trieste, Italy
3 Immunology and Cell Biology Laboratory, Department of Zoology, Cooch Behar Panchanan Barma University, Cooch Behar 736101, West Bengal, India
4 Department of Veterinary Medicine, United Arab Emirates University (UAEU), Al Ain P.O. Box 15551, United Arab Emirates
5 Department of Life Sciences, University of Trieste, 34127 Trieste, Italy
* Correspondence: cagostinis@units.it (C.A.); rbulla@units.it (R.B.); Tel.: +39-040-558-8646 (R.B.); Fax +39-040-558-4023 (R.B.)
† These authors contributed equally to this work.
‡ These authors jointly supervised this work.

Supplementary Figures

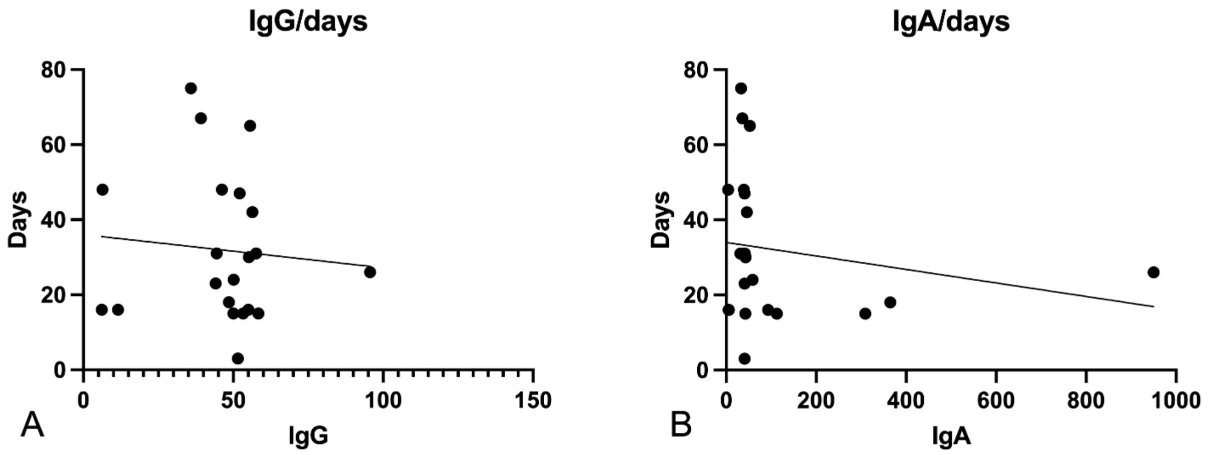

Figure S1. Correlation of IgG (**A**) or IgA (**B**) levels and days after vaccination.

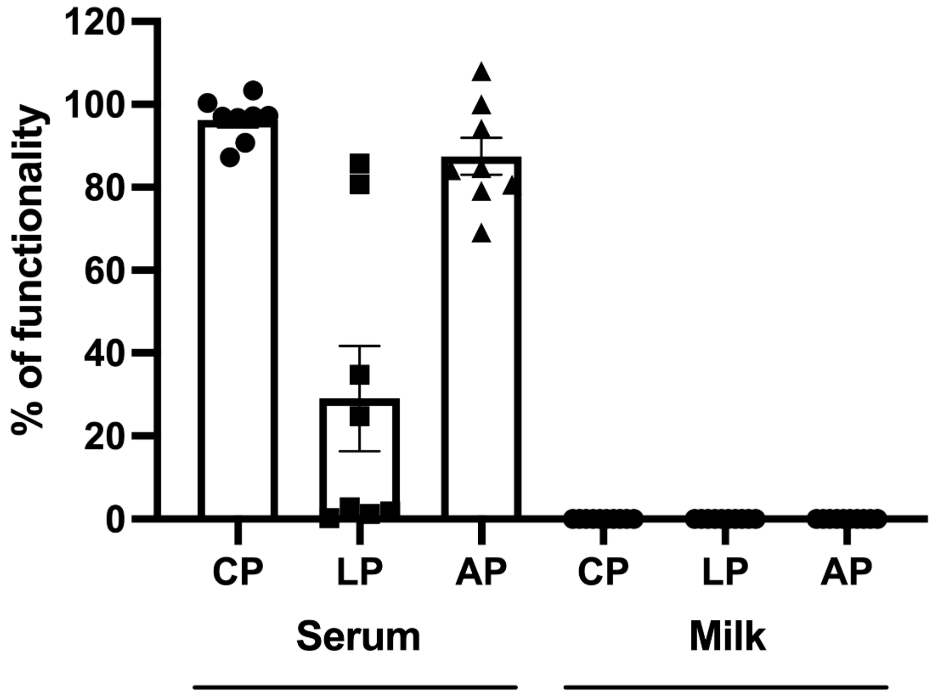

Figure S2. Evaluation of complement (C) functionality in serum and milk samples. Serum and milk samples were tested by Wieslab assay in order to determine their capability of activating the three C pathway: classical (CP), lectin (LP) and alternative (AP). Milk samples failed to determine activation of all the three C cascades, whilst all the sera resulted to be C-sufficient.

Supplementary Tables

Table S1. Characteristics of the women cohort.

Patient	Age (years)	Comorbidity	Previous SARS-CoV-2 Infection	Vaccine Type	Time between Collection and the 2nd Dose of Vaccine (days)	Newborn Age (months)
**1**	32	no	no	Pfizer	31	10
**2**	42	no	no	AstraZeneca	23	31
**3**	44	yes	no	Pfizer	18	3
**4**	31	no	no	AstraZeneca + Pfizer	15	2
**5**	41	no	no	Pfizer	15	13
**6**	30	no	no	AstraZeneca	20	12
**7**	35	yes	no	Pfizer	48	8
**8**	34	yes	no	Moderna	15	2
**9**	31	no	no	Moderna	42	5
**10**	42	no	no	Pfizer	31	20
**11**	31	no	no	Pfizer	48	5
**12**	45	no	no	Pfizer	47	9
**13**	41	no	no	Pfizer	24	19
**14**	34	no	no	Pfizer	75	16
**15**	43	no	no	Pfizer	30	9
**16**	38	no	no	Pfizer	65	4
**17**	34	no	no	Pfizer	67	17
**18**	34	no	yes	Pfizer	26	14
**19**	36	no	yes	Pfizer	15	11
**20**	42	no	yes	Pfizer	17	17
**21**	33	yes	no	AstraZeneca	16	16
**22**	33	no	no	AstraZeneca	16	33

Table S2. Titration of the calibrator used in ELISA assay.

Dilution of Serum Calibrator	Value of Standard Curve	O.D. IgG	O.D. IgA
1:500	0.002	1.165	3.436
1:1000	0.001	1.088	2.452
1:5000	0.0002	0.782	1.027
1:10,000	0.0001	0.652	0.397
1:25,000	0.00004	0.459	0.307
1:50,000	0.00002	0.323	0.174
1:75,000	0.000013	0.311	0.155
1:100,000	0.00001	0.204	0.136
0	0	0.135	0.135

Abbreviations: OD, optical density.
